# On Anthelmintics

**Published:** 1875-11

**Authors:** 


On Anthelmintics.—The *Lancet* says, some investigations have recently been made by M. Heckel into the active part of the pumpkin seeds. These seeds have been much used, of late, for the expulsion of the tapeworm, for which purpose they were employed in the early part of the last century. The mode of their administration has hitherto been to give the bruised seeds in large quantities, suspended in water, the outer envelope only having been removed. About two ounces of the seed was the ordinary dose. It is probable that so large a quantity contains much inert matter. Some recent observations apparently indicate that the active principle is contained only in the embryo. To ascertain whether this is the case was the chief object of M. Heckel's observations. He first administered, in two cases of tænia, about six ounces of the perisperm, tegumentum and testa, a purgative of castor oil having first been administered. The tapeworm was not expelled in any case. In two other cases the membrane surrounding the embryo was given—about half an ounce—preceded and followed by a dose of castor oil. In each case the tapeworm was expelled entire. Subsequent experiments yielded the same result. This membrane was then carefully examined, and found to consist of two membranes, separable by maceration in water. The outer membrane contained a resin in small quantities (one in seventeen,) which M. Heckel believes to be the active agent. He believes that the castor oil acts not only by its purgative effect, but by dissolving the resin and rendering it active. The second membrane contained more chlorophyll than resin. It must not be forgotten that these seeds contain a fixed oil, to which their qualities have been ascribed, and which may be obtained by cold expression from the seeds, in the proportion of half an ounce to a pound. This oil has been used with success, in repeated half-ounce doses, in cases of tænia.—*Medical and Surgical Reporter.*

